# Supplementary material for: Delivery of endocytosed proteins to the cell–division plane requires change of pathway from recycling to secretion
Source: eLife. 2014 Apr 8;3:e02131. doi: 10.7554/eLife.02131 (PMC3979144; doi:10.7554/eLife.02131)
Supplement: Supplementary file 1. — This table summarizes the localization of different vesicle trafficking markers without BFA (1th column) and with BFA in wild-type (Col; 2th column), big3 (3th column), BFA-resistant GNOM (GNR; 4th column) and BFA-resistant GNOM in big3 mutant background (GNR big3; 5th column). Abbreviations: PM, plasma membrane; CP, cell plate; BFA-comp., BFA-compartment. DOI: http://dx.doi.org/10.7554/eLife.02131.016 [file elife02131s001.pdf]

Marker		Col	*big3*	GN^R	GN^R *big3*
	- BFA	+ BFA	+ BFA	+ BFA	+ BFA
**HS::secGFP**	apoplast (Viotti et al., 2010)	apoplast	intracellular aggregate	-	-
**Est>>YFP-SYP132**	PM (this study)	PM	intracellular aggregate	-	-
**Est>>AFVY-RFP**	vacuole (this study)	vacuole	intracellular aggregate	-	-
**BOR1-GFP**	PM (Takano et al., 2005)	PM + BFA-comp.	intracellular aggregate	-	-
**AP1M2-3xHA**	TGN (Park et al., 2013)	BFA-comp.	cytosolic	-	-
**γCOP**	Golgi (Richter at al., 2007)	Golgi	Golgi	-	-
**PIN1 (interphase)**	basal PM (Geldner at al., 2001)	PM + BFA-comp.	PM + BFA-comp.	basal PM	basal PM
**PIN1 (cytokinesis)**	basal PM+CP (Geldner at al., 2001)	PM + CP+BFA-comp.	PM+intracellular aggegate	PM + CP+BFA-comp.	PM+intracellular aggegate
**Est>>PIN1-RFP**	basal PM (this study)	PM + BFA-comp.	intracellular aggregate	basal PM	intracellular aggregate
**KNOLLE**	CP (Lauber at al., 1997)	CP + BFA-comp.	intracellular aggregate	CP + BFA-comp.	intracellular aggregate
**H4::RFP-PEN1**	PM+CP (Reichardt et al., 2011)	PM+CP+BFA-comp.	PM+intracellular aggegate	-	-
**KN::MYC-SYP132**	PM+CP (Reichardt et al., 2011)	PM+CP	PM+intracellular aggegate	-	-

Supplementary Table 1. Localization of vesicle trafficking markers.
This table summarizes the localization of different vesicle trafficking markers without BFA (1th column) and with BFA in wild-type (Col; 2th column), *big3* (3th column), BFA-resistant GNOM (GN^R; 4th column) and BFA-resistant GNOM in *big3* mutant background (GN^R *big3*; 5th column). Abbreviations: PM, plasma membrane; CP, cell plate; BFA-comp., BFA-compartment.
